# Empagliflozin Protects Against Oxidative Stress in the Diabetic Brain by Inducing H_2_S Formation

**DOI:** 10.3390/ph18091259

**Published:** 2025-08-25

**Authors:** Emine Nur Ozbek, Zeynep Elif Yesilyurt Dirican, Medine Makal, Ebru Arioglu Inan, Gunay Yetik-Anacak

**Affiliations:** 1 Department of Pharmacology, Faculty of Pharmacy, Ege University, Izmir 35040, Türkiye; emine.nur.ozbek@ege.edu.tr (E.N.O.); medinemakal@gmail.com (M.M.); 2 Department of Pharmacology, Faculty of Pharmacy, Gazi University, Ankara 06330, Türkiye; zeynepelifyesilyurt@gazi.edu.tr; 3 Department of Pharmacology, Faculty of Pharmacy, Ankara University, Ankara 06560, Türkiye; arioglu@ankara.edu.tr; 4 Department of Pharmacology, Faculty of Pharmacy, Acibadem Mehmet Ali Aydinlar University, Istanbul 34752, Türkiye

**Keywords:** hydrogen sulfide, empagliflozin, oxidative stress, diabetes mellitus, neurodegeneration

## Abstract

**Background:** Hydrogen sulfide (H_2_S) is an endogenously produced gaseous neurotransmitter. H_2_S donors exhibited neuroprotection in oxidative-stress-related disorders in preclinical studies, but odor and short half-lives have limited their clinical use. However, endogenous H_2_S stimulators with antioxidant properties have advantages over H_2_S donors regarding safety and patient compliance. Empagliflozin (EMPA), a sodium–glucose cotransporter-2 (SGLT2) inhibitor widely used in the treatment of diabetes mellitus (DM), exerted similar neuroprotective and antioxidant effects as H_2_S and shares common mechanisms. This study aimed to investigate the role of H_2_S in the antioxidant effects of EMPA in the brain. **Methods:** The effects of EMPA on H_2_S production and reactive oxygen species (ROS) formation were assessed ex vivo in mouse brain under normal conditions and pyrogallol-induced oxidative stress. Moreover, rats were divided into the following four groups: nondiabetic, EMPA-treated nondiabetic, streptozotocin (STZ)-induced diabetic, and EMPA-treated, STZ-induced diabetic. Endogenous H_2_S and ROS levels in the brain were measured using methylene blue and chemiluminescence assays, respectively. **Results:** Ex vivo EMPA treatment significantly increased endogenous H_2_S formation in both healthy and pyrogallol-induced oxidative stress, as well as reduced ROS formation in mouse brain; these effects were significantly reversed by the H_2_S synthesis inhibitor aminooxyacetic acid (AOAA). Oral EMPA administration significantly elevated brain H_2_S levels in both nondiabetic and diabetic rats and reduced ROS formation. These effects were inhibited by AOAA. **Conclusions:** Our study revealed a novel mechanism by which EMPA can reduce oxidative stress in neurodegenerative disorders by triggering H_2_S synthesis in the brain.

## 1. Introduction

Diabetes mellitus (DM) is a complex, chronic metabolic disorder characterized by persistent hyperglycemia due to impaired insulin secretion and/or insulin action. DM can be categorized into two types. Type 1 DM (T1DM) is an autoimmune disease characterized by the immune-mediated destruction of pancreatic β cells, resulting in absolute insulin deficiency and requiring lifelong exogenous insulin therapy [1]. Type 2 DM (T2DM) is characterized predominantly by insulin resistance and relative insulin deficiency, and it is usually associated with genetic predisposition, obesity, and a sedentary lifestyle [2]. In recent years, preclinical and epidemiological studies have reported that hyperglycemia triggers amyloid beta deposition, oxidative stress, and neuroinflammation in the brain, ultimately leading to neurodegeneration and cognitive decline [3]. Impaired insulin signaling in the diabetic brain and the accompanying increase in oxidative stress lead to neuronal loss and cognitive impairment [4]. Therefore, DM has recently been proposed as a risk factor for neurodegenerative diseases [5]. Recently, the term type 3 DM (T3DM) has been defined to refer to brain-specific diabetes, especially for neurodegenerative diseases characterized by cognitive decline, such as Alzheimer’s disease [5,6].

Empagliflozin (EMPA), a selective sodium–glucose co-transporter 2 (SGLT2) inhibitor that has displayed additional cardiovascular and renal benefits, is currently approved and widely used for the management of T2DM. Although not approved for use in T1DM because of concerns over the potential risk of diabetic ketoacidosis, recent evidence from clinical trials suggests that low-dose empagliflozin, when used in conjunction with insulin therapy, might improve glycemic control and metabolic parameters in individuals with T1DM [7,8]. However, the effects of EMPA in the brain in patients with T1DM or related neurodegenerative disorders have not been investigated sufficiently.

Hydrogen sulfide (H_2_S), recognized as the third gasotransmitter alongside nitric oxide and carbon monoxide, is endogenously synthesized from L-cysteine (L-cys) through cystathionine γ-lyase (CSE), cystathionine β-synthase (CBS), and 3-mercaptopyruvate sulfurtransferase (3-MST) enzymes in various tissues, including the brain [9]. Along with its antioxidant, anti-inflammatory, and protective effects in ischemia, H_2_S plays a crucial role in regulating various physiological processes, including neuronal signaling. H_2_S can directly inactivate reactive oxygen species (ROS) or protect cells by activating antioxidant defense systems involved in inflammation, apoptosis, and mitochondrial function, thereby enhancing cellular resistance to oxidative stress [10,11,12]. Prior studies have revealed that treatment with H_2_S donors improved neuronal damage and restored memory and cognition in animal models of neurodegenerative diseases by alleviating oxidative stress and related damage to neurons [10,11,13]. However, because the odor of H_2_S donors can decrease patient compliance and these compounds have short half-lives, agents that increase the endogenous synthesis of H_2_S in the brain are emerging as a new therapeutic approach to support the management of neurodegenerative diseases [14]. Because H_2_S has important therapeutic potential in vascular and neuronal diseases, we investigated H_2_S-inducing drugs in the brain for drug repurposing in neurovascular diseases involving oxidative stress.

Beyond its glucose-lowering effect, EMPA was demonstrated in clinical and preclinical studies to display neuroprotective properties, primarily through its antioxidant and anti-inflammatory effects [15,16,17,18]. Interestingly, the signaling pathways involved in EMPA’s neuroprotective effects through reducing oxidative stress, neuroinflammation, and mitochondrial dysfunction overlap with the downstream mechanisms of H_2_S. Both EMPA and H_2_S activate nuclear factor erythroid 2-related factor 2 (NRF2)/Kelch-like ECH-associated protein 1 (Keap1) [19,20,21,22] and AMP-activated protein kinase AMPK/SIRT1 axes [15,21,23,24]. Additionally, both agents regulate the mTOR pathway [25,26], as well as inhibit NF-κB signaling to suppress neuroinflammation [24,25,27,28]. Another shared neuroprotective mechanism involves the elevation of brain-derived neurotrophic factor (BDNF) levels [29,30] and inhibition of NLRP3 (NOD-, LRR-, and pyrin-domain-containing protein 3) inflammasome activation [17,31]. However, the relationship between EMPA and H_2_S in the brain remains unclear.

The present study aims to investigate whether EMPA could stimulate endogenous H_2_S production and exert antioxidant effects through H_2_S. Therefore, we first tested the effect of EMPA incubation on H_2_S formation ex vivo in mouse brain in the presence/absence of pyrogallol (Pyro)-induced oxidative stress conditions. In addition, the effects of EMPA against oxidative stress in mouse brain and the role of H_2_S in these effects were examined. Further, we investigated the effects of oral EMPA treatment on endogenous H_2_S production and ROS formation in the brains of both healthy and STZ-induced diabetic rat brains.

## 2. Results

### 2.1. *Ex Vivo* Studies

#### 2.1.1. EMPA Increases Endogenous H_2_S Formation Under Healthy and Pyro-Induced Oxidative Stress Conditions in Ex Vivo Mouse Brain 

**Figure 1:**
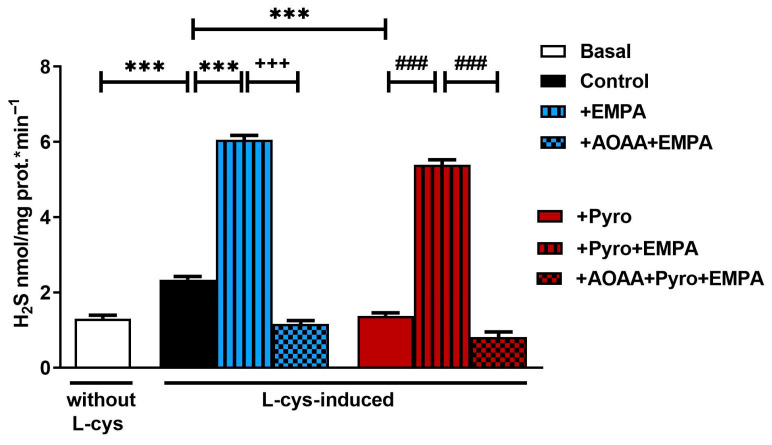
The effect of ex vivo EMPA treatment on l-cys-induced H_2_S formation under healthy and Pyro-induced oxidative stress conditions in the mouse brain homogenates. *** *p* < 0.001, compared to the control; ### *p* < 0.001, compared to +Pyro+EMPA; +++ *p* < 0.001, compared to +EMPA. One-way ANOVA with Bonferroni’s post hoc test (*n* = 6/group).

The addition of l-cys, a substrate of H_2_S synthesis enzymes, induced basal endogenous H_2_S formation in the mouse brain homogenates, thus confirming H_2_S production in the mouse brain as expected (*p* < 0.001; Figure 1). The oxidative-stress-inducing agent Pyro reduced l-cys-induced H_2_S formation in mouse brain compared with the findings in the control group (*p* < 0.001; Figure 1). Although H_2_S formation decreased with oxidative stress, it increased with ex vivo EMPA treatment (1 µM) for 30 min under both healthy and oxidative stress conditions (*p* < 0.001; Figure 1). Moreover, the EMPA-induced augmentation of H_2_S formation was reversed by the H_2_S synthesis inhibitor aminooxyacetic acid (AOAA) under both healthy and Pyro-induced oxidative stress conditions (*p* < 0.001; Figure 1).

#### 2.1.2. EMPA Treatment Protects Against Pyro-Induced Oxidative Stress Effects Through H_2_S in Ex Vivo Mouse Brain

**Figure 2:**
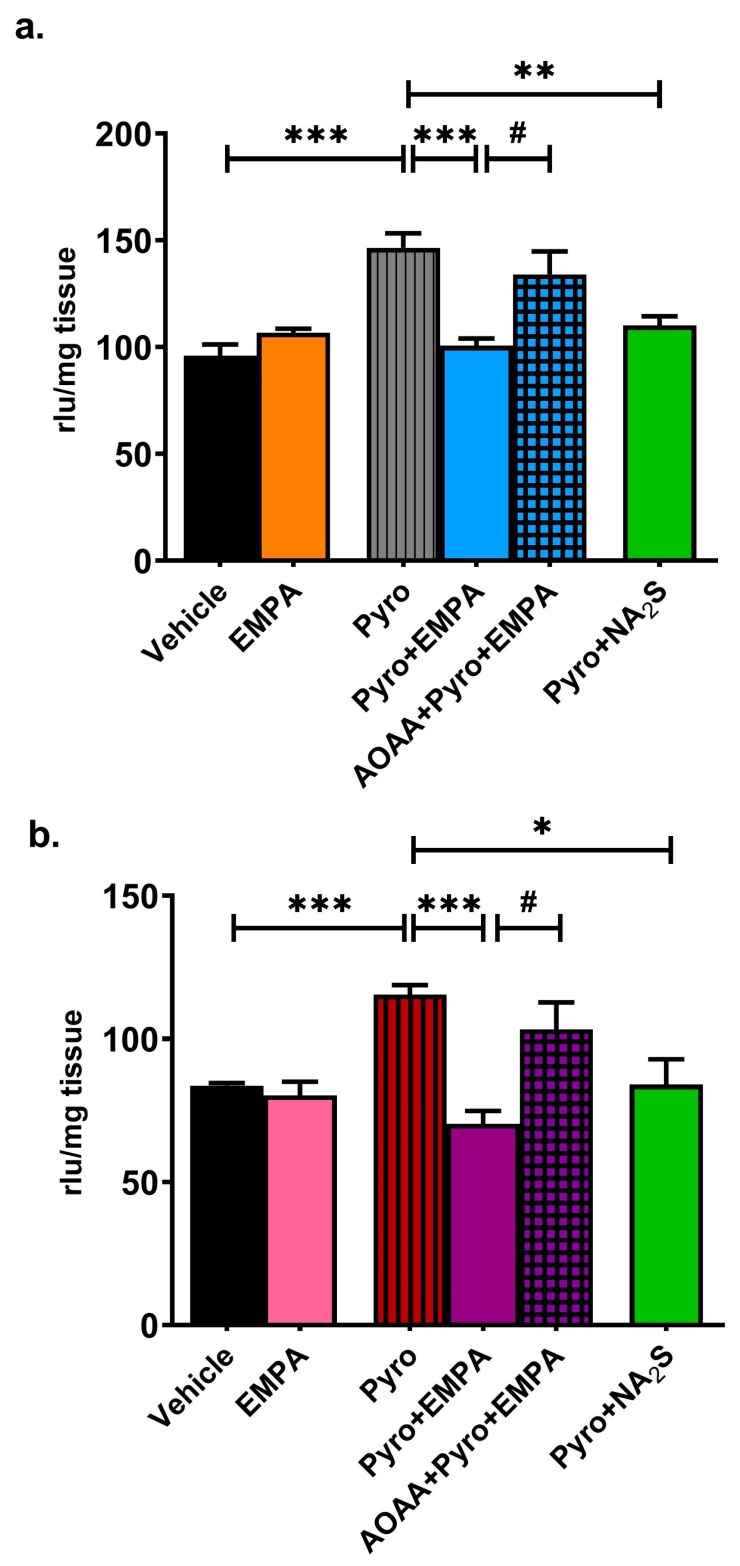
The effect of ex vivo EMPA treatment on oxidative stress induced by Pyro in the mouse brain: (**a**) O_2_^−^ formation: (**b**) other ROS formation. * *p* < 0.05, ** *p* < 0.01, and *** *p* < 0.001, compared to Pyro; # *p* < 0.05, compared to Pyro+EMPA. One-way ANOVA with Bonferroni’s post hoc test (*n* = 6/group).

Pyro increased the levels of both O_2_^−^ radicals and other ROS in the mouse brain compared to the vehicle (*p* < 0.001; Figure 2a,b)**.** No significant difference was observed between the vehicle and control groups (Figure S1). Ex vivo treatment with EMPA (1 µM, 30 min) reduced the formation of Pyro-induced O_2_^−^ and other ROS to the control levels (*p* < 0.001; Figure 2a,b). The antioxidant effect of EMPA was significantly reversed in the presence of AOAA (*p* < 0.05; Figure 2a,b). Moreover, the inhibition of the formation of O_2_^−^ and other ROS by the H_2_S donor Na_2_S confirmed the antioxidant effect of H_2_S in the brain (*p* < 0.01, Figure 2a; *p* < 0.05, Figure 2b; respectively).

### 2.2. *In Vivo* Studies

#### 2.2.1. Effect of In Vivo EMPA Treatment on Weight and Blood Glucose Levels

**Figure 3:**
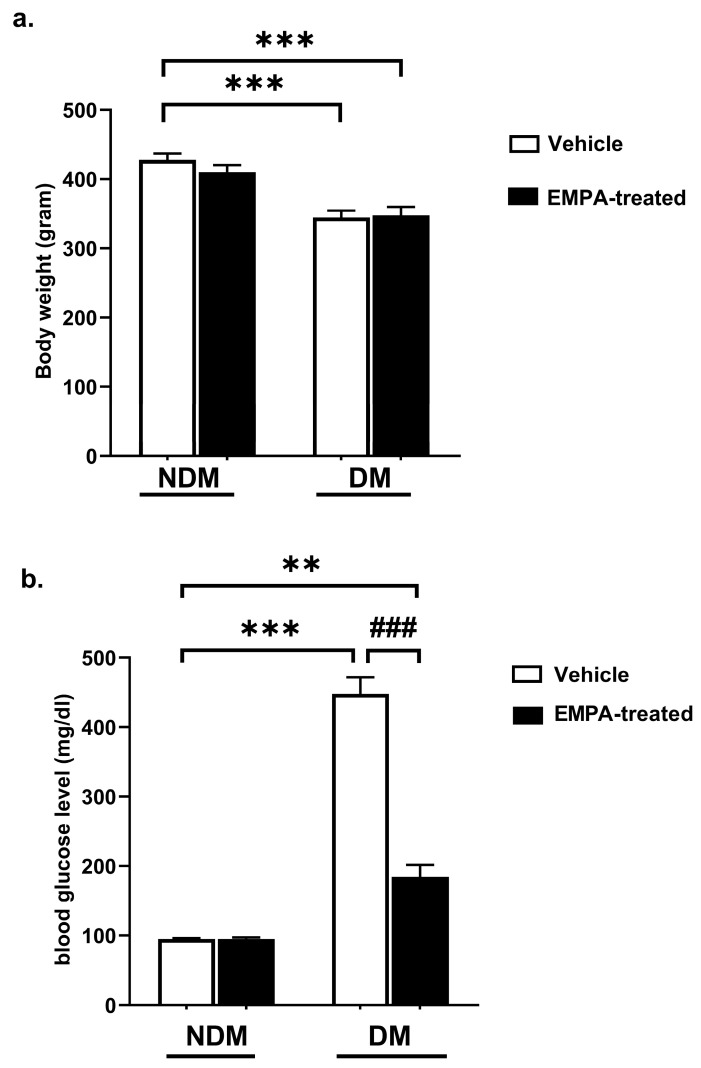
General characteristics of the animals used in the in vivo studies: (**a**) body weight and (**b**) blood glucose levels on the day of sacrifice in NDM (nondiabetic, *n* = 6), EMPA-treated nondiabetic (NDM-EMPA-treated, *n* = 6), diabetic (DM, *n* = 6), and EMPA-treated diabetic rats (DM-EMPA-treated; *n* = 6). ** *p* < 0.01 and *** *p* < 0.001 compared to NDM-vehicle; ### *p* < 0.001 compared to DM-vehicle. One-way ANOVA with Bonferroni’s post hoc test).

Initial body weight did not differ among the groups. At the end of the week 8, body weight was significantly lower in rats with DM compared to NDM rats, as shown in Figure 3a (*p* < 0.001; NDM 427.7 ± 9.04 g, *n* = 6; DM 344.5 ± 10.01 g, *n* = 6). EMPA did not affect DM-induced weight loss (NDM-EMPA-treated: 410.2 ± 9.89 g, *n* = 6; DM-EMPA-treated: 347.3 ± 12.44 g, *n* = 6).

Blood glucose levels were significantly higher in the DM group compared to the NDM group, as shown in Figure 3b (*p* < 0.001; NDM 95.00 ± 1.15 mg/dL, *n* = 6; DM 447.50 ± 24.10 mg/dL, *n* = 6). EMPA treatment in the diabetic rats reduced blood glucose levels statistically significantly compared to the DM group, as shown in Figure 3b (*p* < 0.001; DM-EMPA-treated: 184.5 ± 17.13 mg/dL, *n* = 6; DM 447.50 ± 24.10 mg/dL, *n* = 6). However, EMPA treatment did not alter blood glucose levels in NDM rats (*p* > 0.05; NDM 95.00 ± 1.15 mg/dL, *n* = 6; NDM-EMPA 95.00  ±  2.00 mg/dL, *n* = 6; as shown in Figure 3b).

#### 2.2.2. EMPA Induces H_2_S Formation in the Brains of Rats with STZ-Induced DM

**Figure 4:**
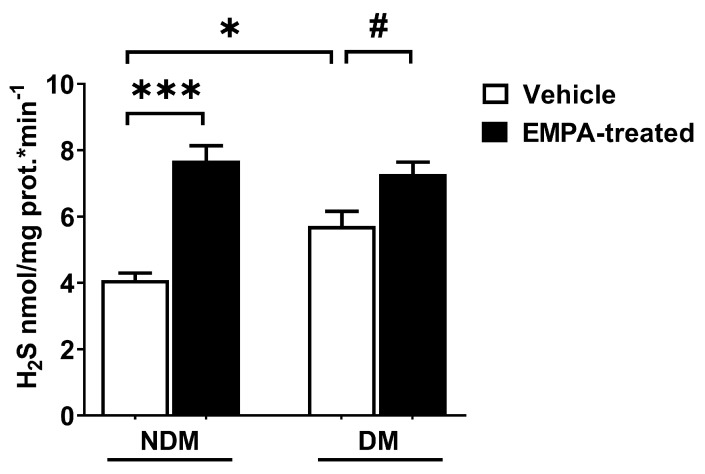
The effect of oral EMPA administration on L-cys-induced H_2_S formation in diabetic (DM) and nondiabetic (NDM) rat brains. * *p* < 0.05 and *** *p* < 0.001, compared to NDM-vehicle; # *p* < 0.05, compared to DM-vehicle. One-way ANOVA with Bonferroni’s post hoc test (*n* = 6/group).

EMPA stimulated L-cys-induced H_2_S synthesis in the brains of both the NDM and DM groups, as shown in Figure 4 (*p* < 0.001 and *p* < 0.05, respectively). In addition, H_2_S formation was higher in the brains of rats with DM compared to NDM, suggesting a compensatory role of H_2_S (*p* < 0.05; Figure 4).

#### 2.2.3. In Vivo EMPA Treatment Exerted Antioxidant Effects Through H_2_S in the Brains of Rats with DM

**Figure 5:**
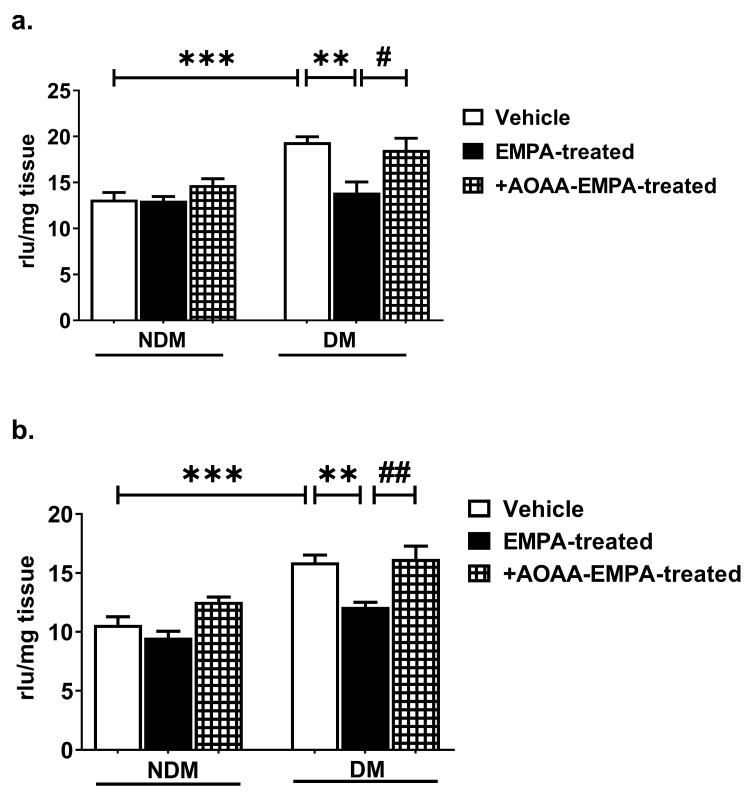
The effect of oral EMPA administration on the formation of (**a**) superoxide radicals and (**b**) other ROS in the brains of nondiabetic (NDM) and diabetic (DM) rats. ** *p* < 0.01 and *** *p* < 0.001, compared to DM-vehicle; # *p* < 0.05 and ## *p* < 0.01, compared to DM-EMPA-treated. One-way ANOVA with Bonferroni’s post hoc test (*n* = 6/group).

DM increased the generation of both O_2_^−^ radicals and other ROS in brain tissues compared to NDM (*p* < 0.001; Figure 5a,b). EMPA treatment normalized this augmentation of ROS levels in rats with DM to levels similar to those in the brains of rats with NDM (*p* < 0.01; Figure 5a,b), and the effect of EMPA in rats with DM was completely reversed by AOAA (*p* < 0.05 and *p* < 0.01, respectively; Figure 5a,b). However, EMPA did not alter ROS levels in the brains of NDM rats, where oxidative stress is not present. These findings suggest that EMPA exerts an antioxidant effect through H_2_S.

## 3. Discussion

SGLT2 inhibitors, which were initially indicated as antidiabetic drugs, have recently gained significant attention in clinical trials and preclinical studies for their therapeutic potential in cardiovascular and neurodegenerative diseases [16,32,33,34]. SGLT2 inhibitors are lipid-soluble drugs that cross the blood–brain barrier and exert neuroprotective effects in the brain [35]. These neuroprotective effects of SGLT2 inhibitors extend their therapeutic potential, especially in cognitive decline associated with T3DM, which is considered as brain diabetes [3].

Similar to EMPA, H_2_S plays a neuroprotective role in the central nervous system. Decreased levels of H_2_S have been reported in the plasma of patients with neurodegenerative diseases such as Alzheimer’s disease and dementia [12,36] and in the brains of animal models [11,37]. Thus, H_2_S donors or drugs that stimulate H_2_S synthesis are suggested to exert therapeutic effects by normalizing decreased levels of H_2_S. Our results suggest that ex vivo treatment with EMPA (1 µM) increases endogenous H_2_S formation in mouse brain under both healthy and Pyro-induced oxidative stress conditions. A concentration of 1 µM was selected in our study to ensure clinical relevance, as the plasma concentration of EMPA reaches 1.87 µM following a 10 mg dose in patients [38]. Although drug metabolism in rodents differs from humans, in the literature the same 10 mg/kg dose has frequently been used in rodents in in vivo studies [17,22,39,40,41,42,43], and a similar concentration of 1 µM has been used in in vitro studies in the literature, which is the same concentration of EMPA reached by 10 mg/kg in human plasma [19,44,45].

Pyro, which induces oxidative stress by generating ROS, is widely used in the hair dye industry, and it is an important toxic agent [46]. Pyro reduces cell growth and induces apoptosis by increasing the production of O_2_^−^ radicals in neuronal cells such as SH-SY5Y and PC12 cells [47,48]. Additionally, in the present study, we found that Pyro-induced oxidative stress reduces endogenous H_2_S levels in mouse brain. This is in line with our findings in pioneering studies demonstrating decreased endogenous H_2_S formation in mouse aorta and lungs following Pyro treatment [49,50].

Moreover, we observed a significant increase in H_2_S levels in the brain homogenates of rats with STZ-induced diabetes. Given the neuroprotective, anti-inflammatory, and antioxidant properties of H_2_S in the nervous system, it can be assumed that brain H_2_S levels increase through a compensatory mechanism. Supporting our findings, increases in CSE mRNA expression in cerebral arteries and H_2_S production in pancreas and liver tissues have been reported in rats with STZ-induced diabetes [51]. Contrary to these findings, other studies observed reduced H_2_S production in the hippocampus [52] and plasma of rats with STZ-induced T1DM [53] and in the circulation of rats with non-obese T1DM [54]. Although these studies highlight the complexity of H_2_S regulation in diabetes, the implications for H_2_S brain levels remain unclear. Regarding neurodegenerative disorders involving oxidative stress, decreased plasma H_2_S levels have been observed in patients with Alzheimer’s disease and dementia [36]; in the brains of APP/PS1 mice, a model of Alzheimer’s disease [55]; in the substantia nigra of a Parkinson’s disease model in rats [11]; and in the blood, cortex, and hippocampus in a traumatic brain injury (TBI) model [56]. However, H_2_S levels returned to baseline levels on day 7 after TBI in the cortex and on day 3 after TBI in the hippocampus. These observations support the findings that H_2_S levels can vary depending on tissue, region, and time [56].

An important result of our study is that oral EMPA (10 mg/kg) administration induced a direct increase in H_2_S formation in the brains of healthy rats and rats with STZ-induced diabetes, which represents T1DM. Because the general H_2_S-synthesis enzyme inhibitor AOAA inhibited the increase in H_2_S formation induced by EMPA, we suggest that H_2_S-producing enzymes might be responsible for the observed effect rather than the H_2_S donor effect of EMPA. Although Wu et al. reported that EMPA treatment (5 µM) did not alter the expression of CSE in HUVECs under basal or d-galactose-stimulated conditions [57], this difference might be due to the tissue-specific expression profiles of H_2_S-producing enzymes. Specifically, we measured H_2_S levels in the brain, in which CBS is the main enzyme responsible for H_2_S production, whereas CSE is predominantly expressed in vascular and endothelial cells [58]. Moreover, although Wu et al. demonstrated that EMPA reduces CSE expression, its effect on CBS expression might differ. In our previous study, we revealed that the expression of H_2_S-producing enzymes was counterbalanced; while CSE expression increased, CBS expression decreased [59]. Moreover, it is known that EMPA activates NRF2 [19], and recently it was reported that NRF2 increases CBS expression [60]. Although we did not investigate it in our study, activation of NRF2 may be the cause of EMPA-induced H_2_S production. In our study, we confirmed that the increased level of H_2_S by EMPA was due to endogenous H_2_S formation, which is inhibited by AOAA, a general inhibitor of H_2_S-synthesizing enzymes including CBS, the main H_2_S-producing enzyme in the brain [60]. Because the induction of endogenous H_2_S production might provide a more controlled strategy for achieving the required H_2_S level without causing more toxicity than H_2_S donors, drugs and other substances that increase endogenous H_2_S formation in the brain have potential to treat neurodegenerative diseases. In addition, agents that increase endogenous H_2_S production could provide better patient compliance versus exogenous H_2_S donors, which have an unpleasant odor. In this manner, EMPA may provide multitarget treatment strategies in diabetes-induced neurodegenerative diseases by inducing endogenous H_2_S synthesis in the brain.

Several studies report the antioxidant effect of EMPA in the brain [19,61,62], but to our knowledge, there is insufficient evidence demonstrating the acute antioxidant effect of EMPA in the brain under in vitro conditions. In our study, we found that ex vivo EMPA treatment reduced Pyro-induced ROS formation in the brain. Because high glucose levels may also induce oxidative stress and the decrease in glucose levels caused by SGLT2 inhibitors might also be involved in the antioxidant effects of EMPA, our findings differs from those of other studies by demonstrating that the acute antioxidant effect of EMPA is independent of its effect on glucose homeostasis. Supporting our result, some other studies show the acute antioxidant effect of EMPA at the same concentration (1 µM) used in our in vitro conditions in cultured cells, such as mesothelial, endothelial, smooth muscle, and coronary artery cells [19,63,64]. However, in these studies, the target tissue/cell was not the brain, and different oxidative stress inducers such as oxidized cholesterol or cyclic stress were used but not Pyro, which has toxicological importance, as it is used to dye hair.

Recent studies have investigated several mechanisms underlying the neuroprotective effects of EMPA in the brain [16,17,41]. Activation of the NRF2/ARE signaling cascade, which causes a reduction in ROS levels by increasing the expression of endogenous antioxidant enzymes [19,22], is suggested as one of the key mechanisms in EMPA-induced antioxidant and neuroprotective effects. EMPA has also been shown to contribute to mitochondrial protection and redox balance by activating the AMPK/SIRT-1/PGC-1α axis in rotenone-induced Parkinson’s disease model [15]. Similarly, in a depression model, EMPA increased GSH and CAT levels, reduced lipid peroxidation, and increased BDNF levels [65]. Furthermore, EMPA has been reported to suppress NF-κB signaling, which leads to a decrease in the expression of pro-inflammatory cytokines, such as TNF-α and IL-1β [27]. Additionally, EMPA upregulates HIF-1α and VEGF, thereby reducing neuronal apoptosis and improving neurobehavioral outcomes in cerebral ischemia/reperfusion injury [43].

Although we did not investigate other signaling pathways involved in the neuroprotective effects of EMPA beyond the gasotransmitter H_2_S in our study, we would like to emphasize H_2_S-induced downstream mechanisms that may contribute to the neuroprotective effect of EMPA and are also enrolled in downstream signaling. Both H_2_S and EMPA (1) modulate the mTOR signaling pathway [24,25]; (2) enhance brain insulin sensitivity, which plays a key role in preserving neuronal function and preventing cognitive decline associated with insulin resistance [66,67]; (3) regulate M1 muscarinic and NMDA receptor activity, which contributes to the maintenance of synaptic function [9,61]; (4) upregulate BDNF, supporting cognitive function [29,30]; (5) inhibit NLRP3 inflammasome activation, a key mediator of neuroinflammation and pyroptosis, which contribute to neuronal damage in metabolic and neurodegenerative diseases [17,31]; and (6) activate the NRF2 antioxidant pathway, which leads to increased transcription of antioxidant response elements (AREs) and upregulation of downstream antioxidant enzymes, such as HO-1, NQO1, SOD, catalase, and GPx [19,20,21]. H_2_S-induced activation of NRF2 occurs through an increase in S-sulfhydration of Keap1, inducing NRF2 dissociation from Keap1, which enhances NRF2 nuclear translocation and expression of antioxidant enzymes to neutralize ROS [21]. In addition, both EMPA [15,21,23,24] and the H_2_S-producing enzyme MPST, located in the mitochondria, improve mitochondrial function and reduce the generation of ROS [68]. Our study may be a pioneer of future research aiming to elucidate the role of these downstream mechanisms of H_2_S in EMPA-induced neuroprotection.

Although SGLT2 inhibitors inhibit the activity of SGLT2 rather than its expression, they also normalize elevated SGLT2 expression to baseline levels in pathological conditions [69,70]. Interestingly, prior research demonstrated that both EMPA and the H_2_S donor GYY4137 can suppress the upregulation of SGLT2 in HUVECs induced by d-gal [57]. These findings suggest that SGLT2 inhibitors have a dual therapeutic benefit in the management of diabetes: they selectively and reversibly inhibit SGLT2 activity and increase H_2_S levels, which, in turn, suppresses SGLT2 expression. This dual effect could explain their success in glycemic control.

In the present study, we demonstrated that the SGLT2 inhibitor EMPA induces endogenous H_2_S formation in the brain and exerts an antioxidant effect through inducing H_2_S formation.. Beyond the blood-glucose-lowering effects of EMPA, we propose the H_2_S pathway as a novel mechanism in the neuroprotective effects of EMPA against oxidative stress in diabetes-related cognitive impairments. We suggest that EMPA could be an advantageous and promising treatment for diabetes, especially T3DM. While our study focuses specifically on the effects of EMPA, it may shed light on the potential relationship between SGLT2 inhibition and H_2_S production. Although the lack of data on other SGLT2 inhibitors in H_2_S formation limits our ability to determine whether the observed effect is drug-specific or represents a broader class effect, since other SGLT2 inhibitors have been shown to activate NRF2 and which in turn have been shown to activate CBS expression, it is worth investigating whether other SGLT2 inhibitors (e.g., dapagliflozin and canagliflozin) can also induce H_2_S formation. This study provides new insights into the potential of EMPA to mitigate the cognitive dysfunction associated with increased oxidative stress in diabetes.

## 4. Materials and Methods

### 4.1. *Ex Vivo* EMPA Treatment of Mouse Brain Tissue

Swiss albino male mice (25–30 g) were obtained from Ege University Animal Center, with ethics committee approval (2023-102). The animals were euthanized via an intraperitoneal injection of ketamine (60 mg/kg) and xylazine (5 mg/kg) for anesthesia, followed by cervical dislocation. Brain tissues were rapidly excised and placed in Krebs solution containing NaCl (118 mM), KCl (4.8 mM), CaCl_2_ (2.5 mM), KH_2_PO_4_ (1.2 mM), NaHCO_3_ (24 mM), glucose (11 mM), and MgSO_4_ (1.2 mM). The brain-tissue sections were incubated with EMPA at a concentration of 1 µM, which was chosen according to previous studies investigating the biological activities of EMPA [19,61,62]. EMPA was dissolved in dimethyl sulfoxide (DMSO) for a stock solution, and the final concentration of DMSO in the experiments was kept below 0.01%.

### 4.2. Induction of Diabetes and Oral Administration of EMPA

Male Sprague–Dawley rats (11–12 weeks old) were obtained from Ankara University Animal Center, with ethics committee approval (2019-4-41) and maintained under a 12 h/12 h light/dark cycle. They were provided ad libitum access to standard chow (Purina Rat Chow; Optima AS, Bolu, Türkiye) and tap water throughout the study period. After one week-long acclimatization period, rats were randomly divided into the following four groups: (1) nondiabetic (NDM, *n* = 6), (2) EMPA-treated nondiabetic (NDM-EMPA, *n* = 6), (3) diabetic (DM, *n* = 6), and (4) EMPA-treated diabetic (DM-EMPA, *n* = 6). Diabetes was induced by a single intraperitoneal injection of streptozotocin (STZ), at a dose of 40 mg/kg, dissolved in citrate buffer (pH 4.5). Nondiabetic rats received only citrate buffer injections. Blood glucose levels were monitored for 3 days post-STZ injection, and rats with levels exceeding 300 mg/dL were classified as diabetic. Two rats each in the diabetic and EMPA-treated diabetic groups required additional STZ injections to achieve the target blood glucose levels.

EMPA was administered for 13–16 weeks after STZ or vehicle injection. Nondiabetic and diabetic rats received 10 mg/kg EMPA suspended in 5 mL of vehicle by oral gavage once daily for 8 weeks. This dosage was selected according to prior studies, in which 10 mg/kg/day was the lowest effective dose investigated [71,72,73]. Nondiabetic rats received distilled water via oral gavage. EMPA treatment was also applied on the day of sacrifice. At the end of the 8-week treatment period, all remaining rats were sacrificed for homogenization.

### 4.3. Measurement of Endogenous H_2_S Levels

#### 4.3.1. Homogenization of Tissues and Measurement of Total Protein Levels

Brains were isolated from the mice and rats in each studied group (approximately 25–30 mg of tissue) and homogenized via cryogenic grinding (Cryomill, Retcsh, Haan, Germany) in phosphate buffer (PPB; pH 7.4) including proteases and phosphatase inhibitors under liquid nitrogen [50]. Total protein concentrations in homogenates were detected by the bicinchoninic acid assay (BCA Kit, BioVision, Milpitas, CA, USA). H_2_S levels were measured in homogenates containing equal amounts of protein (50 µg) in PBS.

#### 4.3.2. Measurement of H_2_S Levels by the Methylene Blue Assay

H_2_S levels were measured in brain homogenates by the methylene blue assay, as previously described [50,74]. Endogenous H_2_S formation was measured by adding the cofactor pyridoxal 5-phosphate (PP, 2 mM) and saline to the homogenates in PPB under basal conditions, and the H_2_S biosynthesis precursor l-cysteine (L-cys, 10 mM, 30 min, 37 °C) was added instead of saline to measure H_2_S levels under stimulated conditions. To investigate the effect of EMPA on endogenous H_2_S formation, 1 µM EMPA was added for 30 min. To induce oxidative stress, homogenates were incubated with Pyro (0.1 mM) for 5 min [49]. The H_2_S synthesis inhibitor AOAA (10 mM, 30 min) was used to investigate the role of H_2_S-producing enzymes in endogenous H_2_S formation [50].

During the incubation period, Na_2_S standard solutions were prepared at concentrations ranging from 250 nM to 3.9 µM to generate a standard curve. PPB was used as the blank. After incubation, all samples, standards, and blanks were treated sequentially with 1% ZnAc_2_ followed by 10% trichloroacetic acid to trap H_2_S and precipitate proteins. Then, N,N-dimethyl-p-phenylenediamine sulfate in 7.2 M HCl and FeCl_3_ in 1.2 M HCl were added, and the mixtures were left in the dark at room temperature for 15 min. The mixtures were then centrifuged at 10,000 rpm for 5 min at 4 °C. Duplicate aliquots of 200 µL from the samples, standards, and blanks were placed on a microplate, and their absorbance at 650 nm was measured using a spectrophotometer (Varioskan, Thermo Scientific, Waltham, MA, USA). The obtained absorbance values were used to determine H_2_S concentrations (nmol) by referring to the standard H_2_S curve (nmol vs. absorbance). The results are expressed as the nmol/mg protein/min [49].

### 4.4. Measurement of Reactive Oxygen Species (ROS) by Chemiluminescence

ROS production was measured by the luminol–lucigenin method, developed by Munzel et al. [75]. Whereas lucigenin selectively detects O_2_^−^ production, luminol measures other ROS, such as OH^−^, H_2_O_2_, and HOCl. After fresh brain tissues were isolated, tissue sections weighing approximately 10 mg were taken and placed in tubes containing 500 µL of PBS–HEPES (pH 7.4) solution.

To investigate the effect of ex vivo EMPA treatment on Pyro-induced oxidative stress, healthy mouse brain tissues were incubated with agents according to the following study groups: (1) control, (2) vehicle (DMSO), (3) Pyro (0.1 mM, 5 min), (4) EMPA (1 µM, 30 min), (5) EMPA (1 µM, 30 min) + Pyro (0.1 mM, 5 min), and (6) AOAA (10 mM, 30 min) + EMPA (1 µM, 30 min) + Pyro (0.1 mM, 5 min).

The effects of oral EMPA treatment on oxidative stress were assessed in rat brain sections in the following groups: nondiabetic, EMPA-treated nondiabetic, diabetic, and EMPA-treated diabetic. In addition to this experimental set, two more groups were designed to elucidate the role of H_2_S in the antioxidant effect of EMPA. Specifically, ex vivo brain tissues of nondiabetic-EMPA or DM-EMPA rats was incubated with AOAA (10 mM, 30 min). At the end of the incubation, the tissues were placed on a microplate containing PBS–HEPES solution, and either lucigenin or luminol (5 µmol/L) was added. Luminometric measurements were performed on the Varioskan device (Thermo Fisher Scientific, Waltham, MA, USA) for 5 min with 1 min intervals. The measured values are given as the relative area under the curve (AUC) of irradiance units per mg of tissue (rlu/mg) [49].

### 4.5. Statistical Analysis

All calculations and graphical presentations were performed using GraphPad Prism 8 (GraphPad, Boston, MA, USA). Significance was accepted at *p* < 0.05. The data are presented as the mean ± SEM (standard error of the mean), and statistical analysis was performed by one-way ANOVA. If there was an interaction between concentrations and treatments, Bonferroni’s multiple comparison post hoc test was used after the ANOVA.

## 5. Conclusions

In conclusion, this study provides the first evidence that the protective effects of the SGLT2 inhibitor EMPA against oxidative stress are mediated by the induction of endogenous H_2_S formation in the brain. Our findings, which demonstrate the relationship between SGLT2 inhibition and H_2_S formation, highlight a novel mechanism regarding the antioxidant and neuroprotective effects of EMPA against diabetes-related cognitive impairment. Given the critical role of H_2_S in neuromodulation, these results suggest that EMPA could offer therapeutic benefits beyond glycemic control and provide a promising strategy for neurodegenerative diseases such as T3DM. Future studies are needed to further investigate the clinical implications of targeting the SGLT2–H_2_S pathway in diabetes-related neurodegeneration.

## Data Availability

Data presented in this study is contained within the article and Supplementary Materials. Further inquiries can be directed to the corresponding author.

## Supplementary Materials

The following supporting information can be downloaded at: https://www.mdpi.com/article/10.3390/ph18091259/s1, Figure S1. The effect of vehicle on oxidative stress induced by Pyrogallol (Pyro) in the mice brain (a) O_2_^−^ formation, (b) other ROS formation. (ns = no significance), *p* > 0.05, control vs. vehicle or Pyro vs. Pyro+vehicle, *** *p* < 0.001, compared to Pyro, ### *p* < 0.001, compared to Pyro+Vehicle, One-Way ANOVA, Bonferroni post hoc test, *n* = 6).

## Abbreviations

The following abbreviations are used in this manuscript:

H_2_S	Hydrogen sulfide
ROS	Reactive oxygen species
T1DM	Type 1 diabetes mellitus
T2DM	Type 2 diabetes mellitus
CSE	Cystathionine γ-lyase
CBS	Cystathionine β-synthase
3-MST	3-mercaptopyruvate sulfurtransferase
T3DM	Type 3 diabetes mellitus
SGLT2	Sodium–glucose co-transporter-2
NRF2	Nuclear factor erythroid 2-related factor
SIRT-1	NAD-dependent deacetylase sirtuin-1
EMPA	Empagliflozin
BDNF	Brain-derived neurotrophic factor
AOAA	Aminooxyacetic acid
