# Supplementary material for: Empagliflozin Protects Against Oxidative Stress in the Diabetic Brain by Inducing H2S Formation
Source: Pharmaceuticals (Basel). 2025 Aug 25;18(9):1259. doi: 10.3390/ph18091259 (PMC12472424; doi:10.3390/ph18091259)
Supplement: Supplementary file 1 [file pharmaceuticals-18-01259-s001.zip › pharmaceuticals-3774483-supplementary.pdf]

*SUPPLEMENTAL MATERIAL*

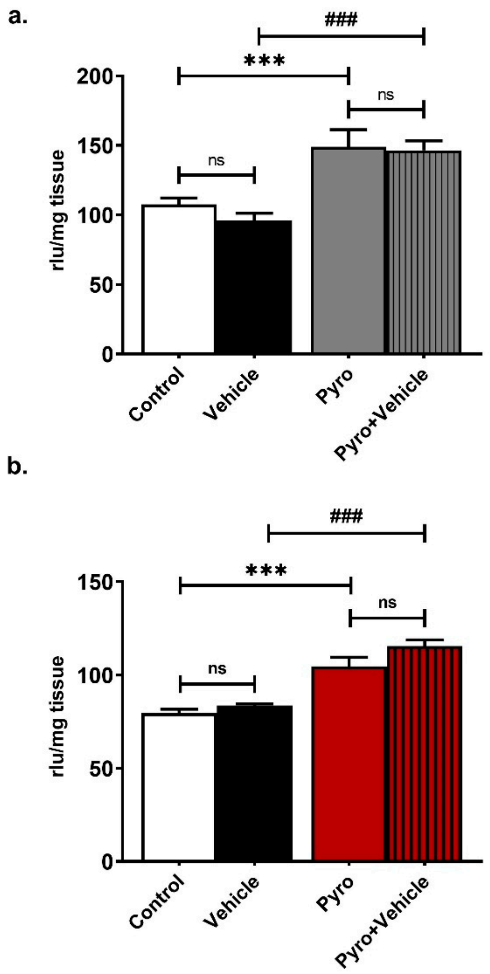


***Figure S1.*** The effect of vehicle on oxidative stress induced by Pyrogallol (Pyro) in the mice brain a) $O_2^-$ formation, b) other ROS formation. (ns=no significance), p >0.05, control vs vehicle or Pyro vs Pyro+vehicle,***p<0.001, compared to Pyro, ###p<0.001, compared to Pyro+Vehicle, One-Way ANOVA, Bonferroni post hoc test, n=6).
